# Accumulation of GSK‐3β in Interneurons Impairs Adult Hippocampal Neurogenesis by Inhibiting GABAergic Transmission

**DOI:** 10.1111/acel.70115

**Published:** 2025-05-26

**Authors:** Fei Liu, Jiu‐Jing Cui, Xiao‐Lin Li, Zeng‐Min Zhang, Shao‐Hua Liang, Yi Sun, Jing‐Min Li, Hong‐Lin Qu, Jing Ye, Qi‐Peng Guo, Quan Zheng, Yong‐Feng Liu

**Affiliations:** ^1^ Department of Human Anatomy, School of Basic Medicine Binzhou Medical University Yantai China; ^2^ School of Rehabilitation Medicine Binzhou Medical University Yantai China; ^3^ Laboratory of Human Anatomy and Histo‐Embryology Binzhou Medical University Yantai China

**Keywords:** adult hippocampal neurogenesis, GABA transmission, glycogen synthase kinase‐3β, interneuron

## Abstract

The activation of glycogen synthase kinase 3β (GSK‐3β) and the deterioration of spatial memory represent prominent pathological and clinical manifestations of Alzheimer's disease (AD). Nevertheless, the precise intrinsic mechanisms linking these pathological features remain poorly elucidated. In this study, we identified significant upregulation of GSK‐3β activity in inhibitory interneurons within the hippocampal dentate gyrus (DG) of 3×Tg‐AD mice. Subsequent investigations demonstrated that targeted overexpression of GSK‐3β in these interneurons triggered aberrant activation of neural stem cells (NSCs), culminating in apoptotic cell death and consequent deficits in adult hippocampal neurogenesis (AHN). Utilizing in vivo fiber‐optic recording techniques, we further established that GSK‐3β overexpression in DG inhibitory interneurons elicited hyperactivation of excitatory neurons, thereby disrupting the excitation–inhibition (E/I) balance within the DG circuitry. Notably, these pathological alterations were ameliorated through chemogenetic suppression of excitatory neuronal activity. Mechanistically, we determined that impaired GABAergic transmission, characterized by reduced GABA release in the DG region, underlies these observed effects. Pharmacological intervention with GABA receptor agonists effectively rescued AHN impairment and attenuated spatial cognitive deficits. Collectively, these findings demonstrate that GSK‐3β overexpression in GABAergic interneurons compromises AHN and promotes NSC apoptosis via disruption of GABAergic signaling, while pharmacological potentiation of GABAergic transmission exerts neuroprotective effects. This study elucidates a previously unrecognized mechanism contributing to AHN impairment in AD and identifies a promising therapeutic target for pro‐neurogenic strategies.

## Graphical abstract

Overexpression of GSK‐3β in DG GABAergic neurons impairs neurogenesis by reducing GABA release, which can be reversed by enhancing GABAergic transmission.

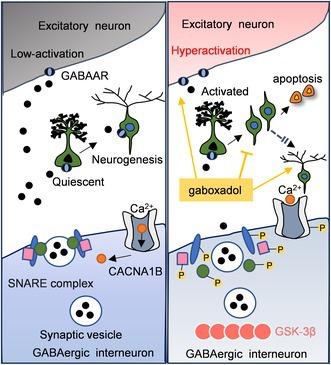

## 1 Introduction

The prevalence of Alzheimer's disease (AD) as a neurodegenerative disease is rising annually in conjunction with the global aging population. The hallmark symptom of AD is progressive cognitive impairment, which can be attributed to a reduction in neuronal count and an overall decline in neuronal function (Braak et al. 1993). In both mice and humans, a subset of the granular cells within the dentate gyrus (DG) is generated postnatally or during adulthood (Jovica et al. 2007; Spalding et al. 2013). Adult hippocampal neurogenesis (AHN) primarily originates from neural stem cells (NSCs) located in the sub‐granular zone (SGZ) region of DG in hippocampus. NSCs are long‐term residents in SGZ and possess capacity for self‐renewal as well as differentiation into various neural and glia cell lineages (Obernier et al. 2018). NSCs receive input from diverse types of neural cells through different neurotransmitters and neuropeptides within a complex neural niche (Zhao et al. 2008). The influence exerted by this neural niche on NSCs proliferation and differentiation ultimately determines the extent of AHN (Goncalves et al. 2016; Kempermann et al. 2015; O'Leary and Cryan 2014; Zhao et al. 2008), which subsequently impacts cognitive function. Therefore, modulating the NSCs fate and enhancing AHN in the brains affected by AD presents a novel strategy for treating this condition.

The hippocampus is among the first brain regions to exhibit pathological changes associated with AD, playing an integral role in higher brain functions such as learning and memory. The normal physiological functions of the hippocampus rely on maintaining a dynamic balance between excitatory and inhibitory efficacy (E/I). Chronically hyperexcitability of neurons within this region can lead to neuronal death when the E/I balance is disrupted (Canter et al. 2016). Glutamate and γ‐aminobutyric acid (GABA) transmission are crucial for ensuring E/I equilibrium within neural networks. Recent studies have shown that GABA‐mediated E/I imbalance affects both proliferation and differentiation processes involving NSCs (Lopatina et al. 2019). Furthermore, heightened activity within hippocampal neural networks during AD pathogenesis (Ramírez‐Toraño et al. 2021) suggests that neuron loss alongside the reduced generation of new neurons may be linked to alteration in GABA transmission. GABAergic interneurons located in the dentate gyrus (DG) portal area govern the excitability of neurons within the DG area through the release of gamma‐minobutyric acid (GABA). This process is crucial for maintaining the excitation–inhibition (E/I) balance in the neural network. GABAergic neurons are subject to remote regulation from other brain regions, and local modulation within the DG area can modify the release of GABA. Such modifications can ultimately lead to an E/I imbalance in the DG area (Bao et al. 2017; Yeh et al. 2018).

Glycogen synthase kinase 3β (GSK‐3β) is a serine and threonine kinase that is highly expressed in the central nervous system. It plays a crucial role in the migration, polarization, and axonal growth of newborn hippocampal neurons during embryonic neural development (Kim et al. 2009). Numerous studies have demonstrated that GSK‐3β activity is elevated in the brains of individuals with AD (Hur and Zhou 2010). The two primary pathological features of AD, namely the formation of fibrillar tangles resulting from hyperphosphorylation of tau proteins and amyloid deposition of Aβ proteins, are both closely associated with the activity of GSK‐3β (Llorens‐Martín Jurado et al. 2014). Additionally, GSK‐3β has been identified as a pivotal enzyme in the regulation of neural progenitor cell homeostasis during mammalian fetal brain development (Kim et al. 2009). Our previous research indicated that early overexpression and activation of GSK‐3β in senescence‐accelerated mice resulted in a depletion of the neural stem cell pool, which subsequently impaired AHN and triggered associated behavioral deficits in senescent mice (Liu et al. 2020). However, whether and how GSK‐3β activation contributes to the decline of AHN through GABA transmission remains unknown.

In this study, we employed an adeno‐associated viral vector (AAV) to simulate the activation of GSK‐3β by overexpressing it specifically in interneurons. We then observed alterations in NSCs and AHN within DG. Furthermore, we investigated the relationship between E/I imbalance and neurogenesis while examining potential underlying causes of E/I imbalance.

## 2 Results

### 2.1 Overexpression of GSK‐3β in GABAergic Interneurons Impaired AHN

**FIGURE 1:**
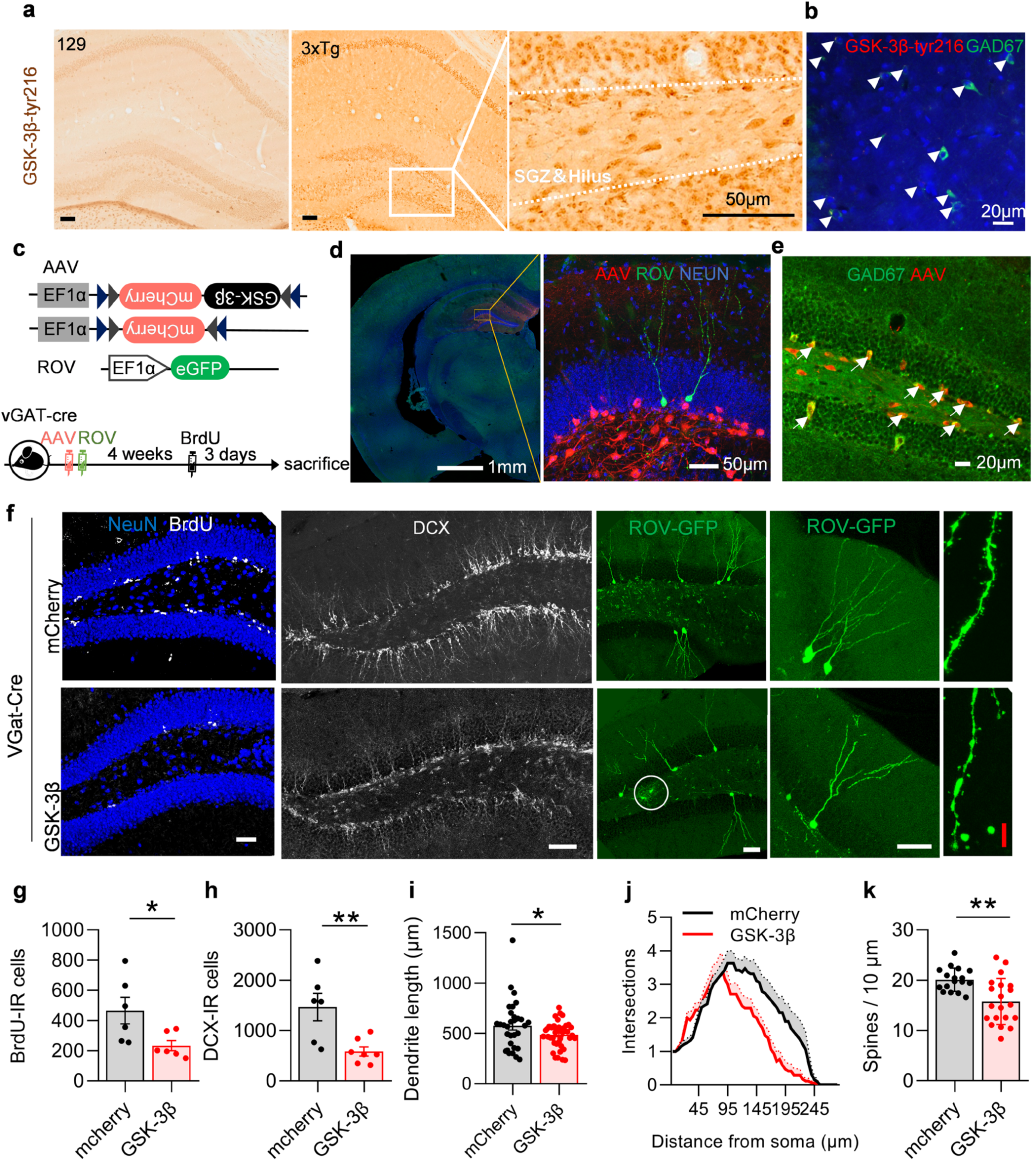
Interneuron‐specific overexpression of GSK‐3β induced AHN deficits. (a) GSK‐3β‐tyr216 aggregated in interneurons within the DG of 3×tg mice compared to vehicle 129 mice. (b) Cells labeled for GSK‐3β‐tyr216 co‐express GAD67. Arrowheads indicate co‐labeled cells. Scale bars were 50 μm (black) or 20 μm (white). (c) Experimental scheme: AAV‐EF1α‐DIO‐GSK‐3β‐mCherry or AAV‐EF1α‐DIO‐mCherry was stereotaxically infused in 2‐month‐old vGAT‐cre mice for specific overexpression of GSK‐3β in interneurons for 4 weeks. Some mice were randomly selected for sacrificing 3 days following a continuous intraperitoneal injection of BrdU. (d) Representative images showing the expression pattern of AAV and ROV in vGAT‐cre mice. The image on the right is an enlarged representation of the square area depicted in the image on the left. Scale bars are indicated in the figure. (e) AAV‐labeled cells co‐express GAD. Arrowheads indicate co‐labeled cells. Scale bar is 20 μm. (f) Representative images showing AHN. Scale bars were 50 μm (white) or 10 μm (red). (g–k) Overexpression of GSK‐3β in GABAergic interneurons reduced the number of BrdU‐ and DCX‐immunoreactive cells (e, f), as well as a decline in the dendritic length (g), complexity (h) and spine density (i) among ROV‐GFP‐labeled newborn neurons. Unpaired *t*‐tests and two‐way ANOVA, *p < 0.05, **p < 0.01. *n* = 6–7 mice (dots) or 17–19 neurons.

To ascertain how the activation of GSK‐3β in AD impairs neural neogenesis, we examined the distribution of activated GSK‐3β within the hilus of DG in 2‐month‐old 3×Tg mice. The results demonstrated that GSK‐3β phosphorylated at tyrosine 216 (GSK‐3β‐tyr216, the activated state of GSK‐3β) aggregated in interneurons (Figure 1a). The majority of those cells labeled by GSK‐3β‐tyr216 were also labeled by GAD67, indicating that they were GABAergic interneurons (Figure 1b).

To investigate the role of GSK‐3β activation in inhibitory interneurons, we constructed the AAV‐EF1a‐DIO‐GSK‐3β‐mCherry virus and its control virus AAV‐EF1a‐DIO‐mCherry (Figure 1c). Each of the two viruses was injected into the DG region of vGAT‐cre mice (which specifically express the cre gene in GABAergic interneurons) to specifically overexpress GSK‐3β in GABAergic interneurons to mimic GSK‐3β activation (Figure 1d). Cells infected by the viruses co‐localized with those labeled by GAD67, further indicating their classification as inhibitory interneurons (Figure 1e).

One month after virus injection, mice were sacrificed following an intraperitoneal injection of bromodeoxyuridine (BrdU, 50 mg/kg) for five consecutive days to assess cell proliferation. Additionally, the ROV‐GFP virus was injected into the DG area to trace developmental progression among newborn neurons (Figure 1c). Doublecortin (DCX) is exclusively expressed in newborn neurons and is used to quantify their number. Results demonstrated a marked reduction in both BrdU‐labeled proliferating neurons and DCX‐labeled newborn neurons within the DG from mice expressing elevated levels of GSK‐3β compared to control mice (Figure 1f–h). Additionally, the length and complexity of the dendrites of the ROV virus‐labeled newborn neurons were diminished, as well as the density of the dendritic spines, indicating that the newborn neurons were poorly developed (Figure 1f,i–k).

### 2.2 Overexpression of GSK‐3β in GABAergic Interneurons Activated NSCs and Promoted Apoptosis

**FIGURE 2:**
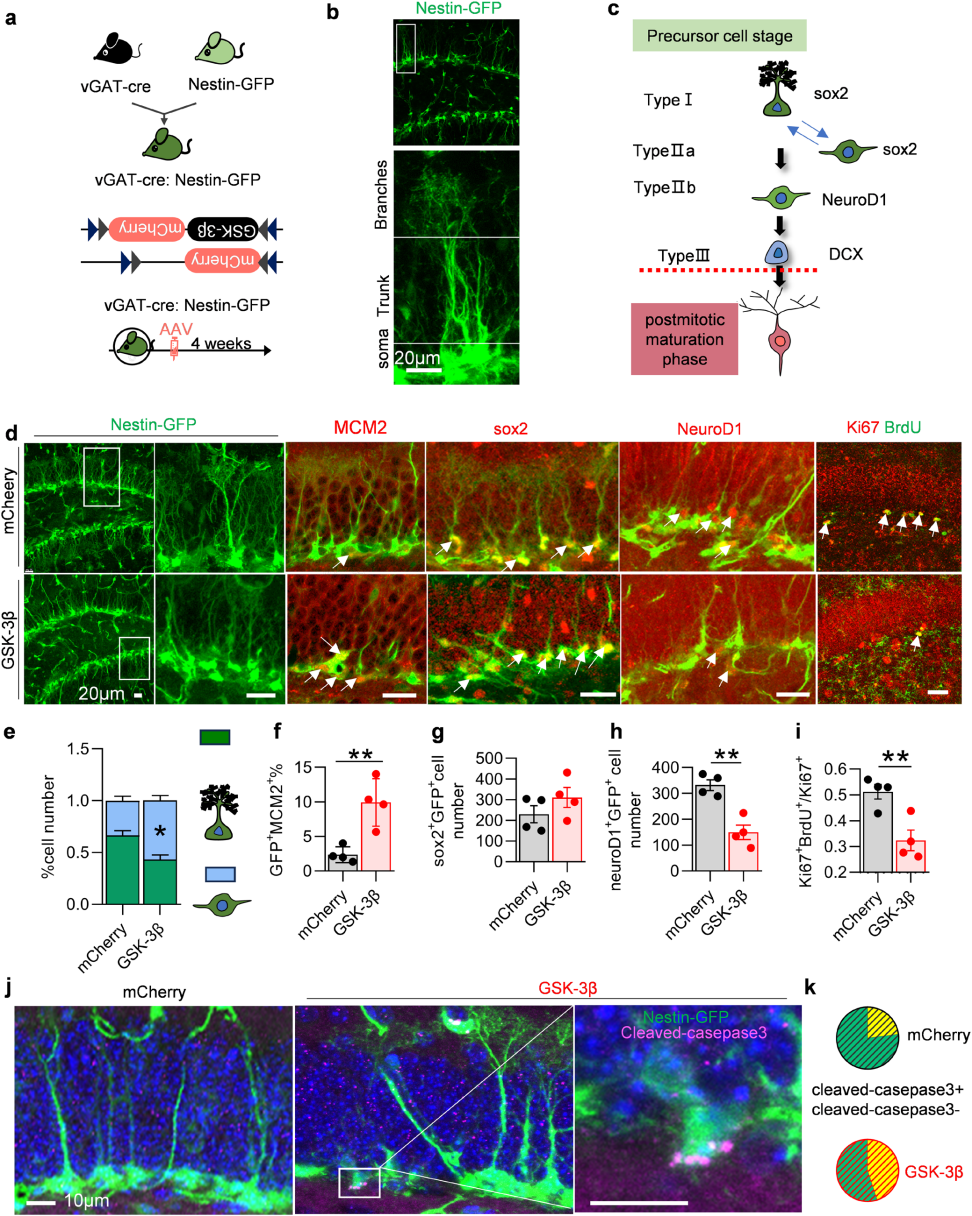
Interneuron‐specific overexpression of GSK‐3β activated NSCs and increased NSCs apoptosis. (a) Experimental scheme: vGAT‐cre: Nestin‐GFP mice were generated by crossbreeding vGAT‐cre with Nestin‐GFP strains. AAV was stereotaxically infused to selectively overexpress GSK‐3β in GABAergic interneurons. (b) A representative image showing the typical morphology of quiescent NSCs. Scale bar, 20 μm. (c) A cartoon illustrating the various stages of differentiation from NSCs to newborn neurons. (d) Representative images showing the NSCs in vGAT‐Cre: Nestin‐GFP mice. (e–i) Overexpression of GSK‐3β in GABAergic interneurons activated NSCs: Morphology of GFP‐labeled Nestin^+^ cells was altered with an increased proportion of type II cells (e). The number of GFP positive cells co‐labeled with MCM2 increased (f), whereas the count of GFP positive cells co‐labeled with Sox2 remained unchanged (g). Conversely the number of GFP positive cells co‐labeled with NeuroD1 decreased (h). Meanwhile, the number of cells co‐labeled with Ki67 and BrdU decreased (i). (j, k) GSK‐3β overexpression in GABAergic interneurons led to enhanced NSCs apoptosis and increased proportion of GFP positive cells co‐labeled with cleaved casepase3 (j), as illustrated in the representative image (k). Unpaired *t*‐tests and two‐way ANOVA, *p < 0.05, **p < 0.01. *n* = 4 mice (dots).

To examine the impact of specific overexpression of GSK‐3β in interneurons on the AHN, we crossed vGAT‐cre mice with Nestin‐GFP mice (which express green fluorescent protein [GFP] proteins in neural progenitor cells, thereby indicating their development) to obtain vGAT‐cre:Nestin‐GFP mice (Figure 2a). This mouse line expresses Cre recombinase and has GFP specifically labeled on the Nestin protein for visualizing and tracking Nestin‐expressing cells (Figure S1). Subsequently, we injected AAV‐EF1a‐DIO‐GSK‐3β‐mCherry virus and its control virus, AAV‐EF1a‐DIO‐mCherry, into the DG region of these vGAT‐cre:Nestin‐GFP mice. The GFP‐labeled neural stem cells were analyzed 1 month later.

NSCs expressing Nestin labeled by GFP can be categorized into three distinct types based on their differentiation stages. Type I NSCs, radial glia‐like cells, are characterized by neuronal dendrites that terminate in a fog‐line structure (Figure 2b). This type of cell plays a crucial role in the adult development of both neurons and glial cells (Urban et al. 2019). Type II intermediate progenitor cells (IPCs), which also express Nestin, exhibit a loss of radial dendrites during their morphological development (Kempermann 2015). IPCs can further be subdivided into two subtypes, type IIa and type IIb (Hodge et al. 2008). Type IIa cells have been observed to possess three potential fates: regression to type I cells, differentiation into mature neurons or glial cells, or undergoing apoptosis (Kempermann et al. 2015). In contrast to type I and type IIa cells that express Sox2 protein, type IIb IPCs have been observed as precursors for newborn neurons (Barbara et al. 2006). These precursor cells initially express the transcription factor NeuroD1 before maturing into fully functional neurons (Figure 2c).

Following the overexpression of GSK‐3β in intermediate inhibitory neurons, the number of Nestin‐positive cells remained unchanged. However, alterations in cell morphology were observed, characterized by shortened and reduced fog‐like dendrites (Figure 2d). The proportion of morphologically altered Nestin‐positive cells increased significantly (Figure 2e). Additionally, the elevated expression of MCM2 (mitotic marker) in Nestin‐positive cells indicated an increase in type II IPCs (Figure 2f). It is noteworthy that both type II IPCs express Sox2, yet only type IIb neural progenitors express NeuroD1 (a marker for early neurons). Expression of NeuroD1 was significantly reduced in the overexpressing GSK‐3β group (Figure 2g,h), suggesting that fewer type IIb cells ultimately differentiate into neurons. What is the fate of Type IIa cells? In the subsequent experiment, the proliferating cells in the DG region were labeled with Ki67 and the surviving cells in the DG region were labeled with BrdU. In the group with overexpression of GSK‐3β, the number of cells co‐labeled with Ki67 and BrdU decreased, which indicates that the survival rate of the proliferated cells was reduced (Figure 2d,i). To further explore the fate of Type II Nestin‐positive cells, the experiment involved the labeling of Nestin‐positive cells with cleaved caspase3, followed by the counting of the number of Type II Nestin‐positive cells co‐labeled with cleaved caspase3. The results obtained demonstrated that the number of cells that were co‐labeled increased in the overexpressing GSK‐3β group, indicating an increased rate of apoptosis (Figure 2j,k).

### 2.3 Overexpression of GSK‐3β in GABAergic Interneurons Enhanced the Excitability of DG

To investigate the molecular mechanisms by which GSK‐3β regulates NSCs, we conducted proteomic and phosphoproteomic analyses on proteins extracted from the DG region. A total of 53/7858 proteins and 327/7844 phosphopeptides in 251/2246 proteins were quantified to be statistically up‐ or downregulated (using a fold change threshold of 1.2) in our proteomic and phosphoproteomic analyses (Figure S2a). The integrated analysis of both the proteome and phosphorylated proteome revealed significant alterations in a substantial number of proteins and phosphorylation sites associated with synaptic transmission (Figure S2b).

**FIGURE 3:**
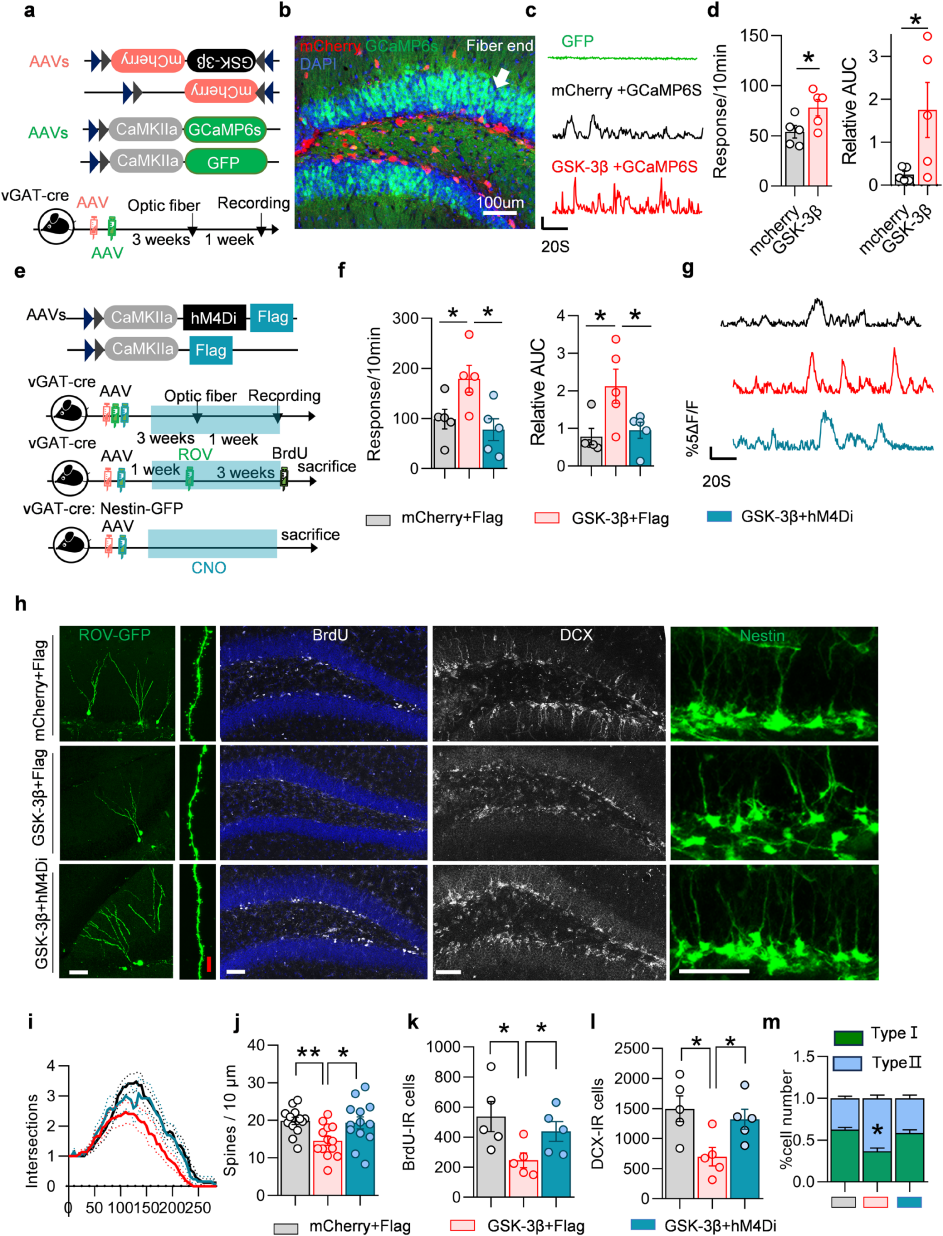
Interneuron‐specific overexpression of GSK‐3β induced network hyperactivation. (a) Experimental procedure of AAVs injection and in vivo optical fiber recording. (b) The representative image showed the pattern of virus infection. Arrows indicate the position of optic fiber. (c, d) Specific GSK‐3β overexpression enhanced calcium response in DG excitatory neurons, as evidenced by an increased number of calcium response (d, left) and a greater area under the Δ*F*/*F* curve (AUC) (d, right). Representative Δ*F*/*F* signals are presented in (c). (e) Experimental procedure for AAVs injection and in vivo optical fiber recording. (f, g) Administration of hM4Di + CNO for a month resulted in a decreased in both the number of calcium response (f, left) and the area under the Δ*F*/*F* curve (AUC) (f, right). Representative Δ*F*/*F* signals are shown in (g). (h) Representative images depicting AHN. Scale bars were 50 μm (white) or 10 μm (red). (i–l) Chemogenetic inhibition partially rescued the AHN deficits induced by interneuron GSK‐3β overexpression. Dendritic complexity (i) and dendritic spine density (j) showed recovery in ROV‐labeled newborn neurons; this recovery was observed within BrdU‐ and DCX‐labeled cell numbers (k, l). (m) The increased proportion of type II cells showed a similar recovery. Unpaired *t*‐tests and two‐way ANOVA, *p < 0.05, **p < 0.01. *n* = 5 mice (dots) or 13–17 neurons.

Impairment in synaptic transmission can lead to E/I imbalance, thereby affecting the transition between quiescent and active states of NSCs (Lopatina et al. 2019). Does overexpression of GSK‐3β influence the excitability within the DG region? The ability of cells to release calcium ions is closely related to their excitability. To assess this, AAV‐CamkII‐Gcamp6s was injected into the DG region of mice, allowing us to measure subsequent calcium response indicative of activity among adjacent excitatory neurons (Figure 3a,b). Compared to control mice infected with mCherry or GFP, specific overexpression of GSK‐3β in interneurons resulted in a marked enhancement in calcium response within the DG (with 5% Δ*F*/*F* as the threshold) (Figure 3c,d), suggesting an increase in excitatory neuron activity.

Furthermore, chemical agents were utilized to chronically inhibit the excitatory neurons. The AAV‐EF1a‐DIO‐GSK‐3β‐mCherry and AAV‐CamkII‐hM4di‐Flag were co‐injected into the DG of vGAT‐Cre or vGAT‐cre:Nestin‐GFP mice (Figure 3e–g). Clozapine N‐oxide (CNO) was administered continuously via drinking water (1.25 mg/mL) for a period of 6 weeks, as previously described (Yeh et al. 2018). The results indicated that chemical inhibition of excitatory neurons markedly enhanced the development of the AHN in a model of dysplasia caused by specific overexpression of GSK‐3β. Additionally, this intervention resulted in an increase in dendrite complexity and dendrite spine density among ROV‐labeled neonatal neurons (Figure 3h–j). The reduction in BrdU‐ and DCX‐labeled cells was also alleviated by chemical inhibition (Figure 3h,k,l). The ratio of type II Nestin^+^ NSCs within the chemical inhibition group was decreased while the ratio of type I NSCs increased (Figure 3h,m).

### 2.4 Overexpression of GSK‐3β in GABAergic Interneurons Resulted in Impairment of GABA Transmission; GABA Receptor Agonists Ameliorate Neurodevelopment

**FIGURE 4:**
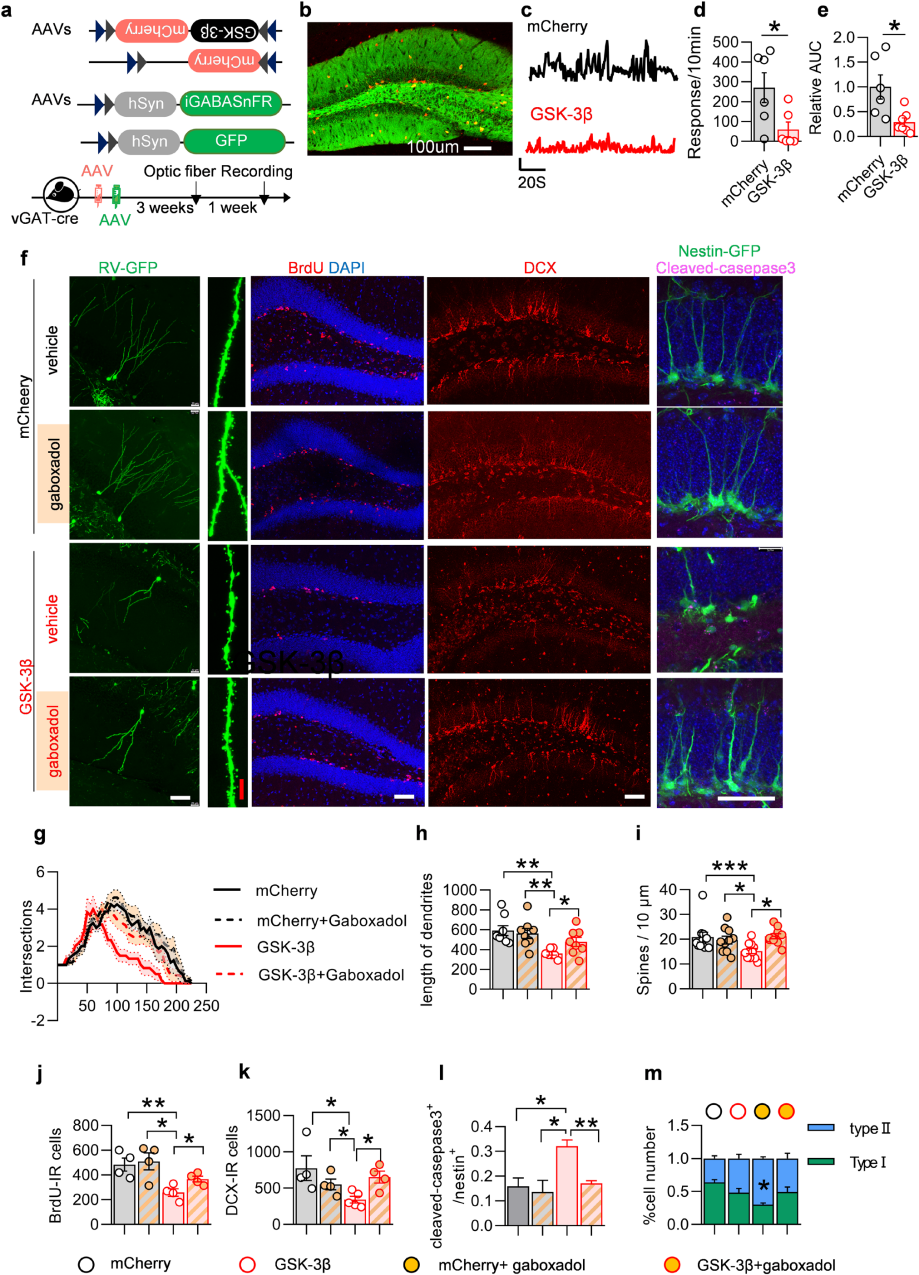
Interneuron‐specific overexpression of GSK‐3β attenuated GABAergic transmission. (a) Experimental procedure for AAVs injection and in vivo optic fiber recording. (b) The representative image showed the pattern of virus infection. (c–e) Interneuron GSK‐3β overexpression attenuated GABA responses (take 5% Δ*F*/*F* as threshold) in DG, compared to the mCherry control group. Representative Δ*F*/*F* signals of iGABASnFR are shown in (c). Unpaired *t*‐tests, *n* = 6 mice in each group, *p < 0.05. (f) Representative images showing AHN. Scale bars were 50 μm (white) or 10 μm (red). (g–k) Gaboxadol rescued AHN deficits caused by GSK‐3β overexpression, as evidenced by the recovery of dendritic complexity (g), dendritic length (h) and dendritic spine density (i) in ROV‐labeled newborn neurons, along with recovery in the number of BrdU (j) and DCX (k) labeled cells. (l) The number of cells co‐labeled with GFP and cleaved‐casepase3 was reduced. (m) The morphology of the GFP‐labeled Nestin^+^ cells showed improvement. The proportion of type II IPCs was restored. Unpaired *t*‐tests and two‐way ANOVA, *p < 0.05, **p < 0.01, ***p < 0.001. *n* = 4–6 mice (dots) or 6–9 neurons.

E/I imbalance in the DG region is associated with the transmission of GABA (Bao et al. 2017; Zheng et al. 2020). The release and instantaneous distribution of GABA in DG was detected using a fluorescent GABA probe (Figure 4a,b). It was observed that the release of GABA was significantly reduced following GSK‐3β overexpression in interneurons (Figure 4c–e). Results from the immunofluorescence and Western blotting experiments indicated no alteration in GABA synthesis within the DG (Figure S3a,b). Furthermore, overexpression of GSK‐3β did not affect intracellular levels of GABA in interneurons (Figure S3c). The expression levels of proteins related to GABA synthesis (GAD65/67), transport (vGAT), release mechanisms (synaptotagmin1, synapsin1, VAMP2), reuptake process (GAT1), and other associated pathways remained unchanged as well (Figure S3d,e).

What factors contribute to the reduced GABA release observed using the GABA fluorescent probe? GSK‐3β is a well‐characterized kinase known for its extensive array of phosphorylated substrates. Additionally, a phosphorylomic analysis of DG revealed that a total of 327 phosphorylation sites across 251 proteins were altered. Among these, 218 exhibited increased levels of phosphorylation, while 109 showed decreased levels (Figure S2a, below). The aforementioned altered phosphorylation sites are implicated in various biological processes, including synaptic transmission, assembly, synaptic plasticity, calcium ion transport, and vesicle regulation (Figure S4a). Concurrently, the results of the Kyoto Encyclopedia of Genes and Genomes (KEGG) analysis indicated that notable changes were observed in 142 pathways. Notably, the GABAergic synaptic pathway was significantly diminished among these pathways (Figure S4b,c) Further investigations revealed that the phosphorylation of certain presynaptic proteins was significantly enhanced at serine and threonine residues. Similar modifications were observed in calcium channel proteins (Figure S4d,e). These proteins play crucial roles in the release of synaptic vesicles.

Further studies revealed that phosphorylation at serine residues of the CACNA1 protein was significantly enhanced (Figure S4e). This protein is a presynaptic N‐type voltage‐dependent calcium channel (VDCC), which mediates Ca^2+^ influx during vesicle release and plays a critical role in vesicle membrane fusion and release. Enhanced phosphorylation of this protein markedly inhibits calcium channel activity, thereby suppressing vesicle release. Phosphoproteomic analysis also showed that phosphorylation at serine and threonine residues of key components of the SNARE complex, such as synapsin1 and syntaxin, was significantly increased (Figure S4d). This complex is essential for directing vesicles to the presynaptic membrane and triggering vesicle fusion and release (Jahn et al. 2023). Phosphorylation of its key components inhibits the formation of the SNARE complex (Snyder et al. 2006) and suppresses vesicle fusion and release.

To further substantiate the hypothesis that a reduction in GABA release is a contributing factor to neurogenesis injury, we conducted the following experiment. The GABA receptor agonist gaboxadol was administered subcutaneously to mice overexpressing GSK‐3β to promote the utilization of GABAergic neurotransmitters by activating the GABAA receptor. Gaboxadol was administered on a biweekly basis for 30 days. The results indicated that the damage to AHN caused by the overexpression of GSK‐3β was ameliorated (Figure 4f). Similarly, the morphological damage of ROV‐labeled newborn neurons was also improved with an increase in the complexity of damaged dendrites (Figure 4g), dendrite length (Figure 4h), and an increase in the density of dendritic spines (Figure 4i). Additionally, the number of newborn neurons labeled by DCX and BrdU was increased by gaboxadol (Figure 4j,k). The number of GFP‐labeled Nestin^+^ cells with a fog dendrite morphology increased, while the proportion of type I NSC increased and the proportion of type II NSC decreased (Figure 4m). The number of Cleaved‐Caspase3‐labeled type II NSCs was found to decrease (Figure 4l).

### 2.5 GABA Receptor Agonists Ameliorate Contextual Memory Impairments Caused by GSK‐3β Overexpression

**FIGURE 5:**
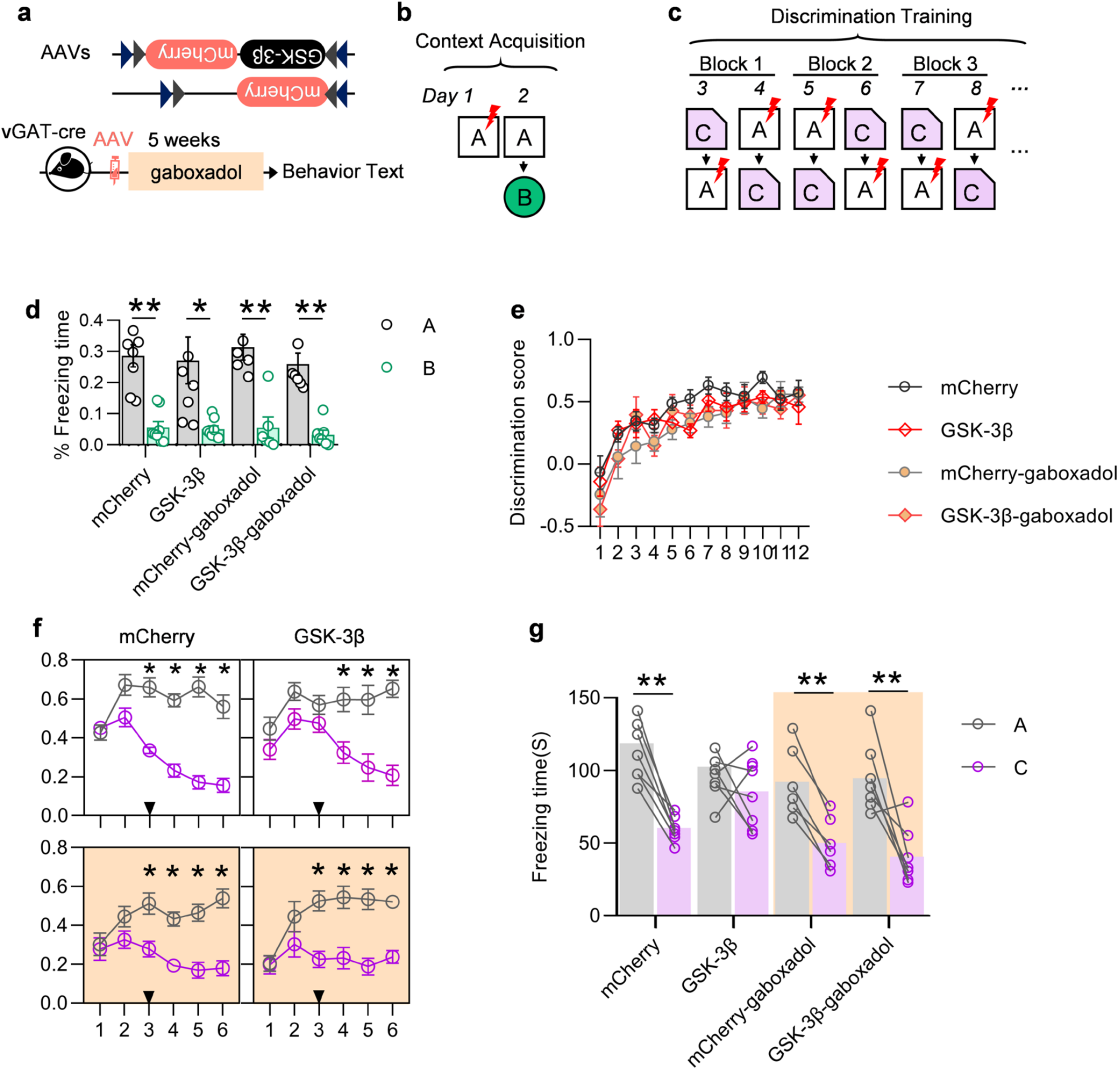
GABA receptor agonist reverses GSK‐3β overexpression induced learning and memory deficits. (a) Experimental procedure: VGAT‐cre mice (2 months old) received AAV virus injections and THIP treatment for 5 weeks. (b) Behavioral training and testing protocol for contextual learning and pattern separation. (c) Mice effectively distinguished environment A from environment B (*p < 0.05, unpaired *t*‐test; *n* = 6–8/group). (d) Alternating training in environment A and environment C. (e) Mice gradually improved discrimination between the two environments, with increasing preference scores (*p < 0.05, unpaired *t*‐test; *n* = 6–8/group). (f) GSK‐3β‐overexpressing mice showed slower learning in distinguishing environment A from environment C. By the third block, controls reduced freezing in environment C, while overexpression mice showed no difference. THIP restored discrimination ability. (g) Freezing times in environment A and environment C during the third block (*p < 0.05, **p < 0.01; repeated measures ANOVA, Tukey's test, or paired *t*‐test; *n* = 6–8/group).

New born neurons have been shown to play a pivotal role in the context of hippocampus‐related learning and memory (Chenn and Walsh 2002). The present study set out to examine the ability of mice to discriminate between different environments and to distinguish patterns (Figure 5b,d). One day after the administration of electrical stimulation, no significant difference was observed in the mice's ability to discriminate between environments A and B (Figure 5c). However, after a 12‐day training period, the mice demonstrated a gradual ability to discriminate between environment A and environment C (Figure 5e). However, when discriminating between environments with less distinguishing features (environments A and C, matching and mismatching plantar electric shocks, respectively) and pattern separation (Patter separation), control mice demonstrated a faster learning capability to discriminate between environments A and C. At the third block of training (i.e., days 7–8), the fear‐like behaviors exhibited by control mice in environment C were significantly less than those in environment A, whereas GSK‐3β‐overexpressing mice could not discriminate between environments A and C until the fourth block of training (i.e., days 9–10), and this ability to discriminate between environments was improved by subcutaneous injection of THIP (Figure 5f,g).

## 3 Discussion

### 3.1 Excessive Activation of GSK‐3β in Interneurons Impaired AHN

GSK‐3β plays a crucial role in regulating a multitude of signaling pathways, thereby participating in a wide range of cellular processes (Jope and Johnson 2004). Both GSK‐3β and its upstream and downstream regulators are essential for neural neogenesis and development (Hur and Zhou 2010). Excessive enhancement of GSK‐3β activity has been shown to impair neuronal dendritic development and synaptic plasticity (Llorens‐Martin et al. 2013; Zhu et al. 2007), with such impairments being commonly observed in AD pathology (Kwok et al. 2008; Simone et al. 2020). The present study demonstrated that aberrant activation of GSK‐3β is evident in GABAergic interneurons located within the DG area of AD model mice. GABAergic interneurons represent a particularly vulnerable subset concerning AD‐related factors (Laure et al. 2012; Li et al. 2009; Palop and Mucke 2016). A significant decline in the number of these interneurons has been reported both in the brains of AD patients and within the hippocampal regions of AD mouse models (Brady and Mufson 1997; Cattaud et al. 2018; Slavica et al. 2012). In AD mice, the aggregation of phosphorylated tau and the increase in Aβ are closely linked to the function of GABAergic interneurons and the process of GABA transmission (Palop and Mucke 2016; Zheng et al. 2020). ApoE4, a highly pathogenic gene for AD, disrupts neurogenesis via the GABAergic signaling pathway, negatively affecting normal brain development and function maintenance (Li et al. 2009).

In this study, we demonstrated that GSK‐3β accumulation detrimentally impacts adult hippocampal neurogenesis (AHN), closely associated with neural stem cell (NSC) activation. As a previous study shows (Boldrini et al. 2018), type I NSC dynamic activation is essential for maintaining the stem cell pool, and the fate of activated NSCs determines AHN quantity. Although NSCs can differentiate into neurons and glial cells (Sierra et al. 2015), in our experiment, NSCs did not transform into glial cells. Instead, apoptosis occurred in type II NSCs. Previous studies (Lipton and Rosenberg 1994; Reynolds and Hastings 1995) have shown that glutamate toxicity causes oxidative stress and calcium overload, activating apoptotic pathways. This leads to neural precursor cell apoptosis, reducing the number of cells for mature neuron differentiation and hampering neural neogenesis. Notably, we found that GSK‐3β abnormally accumulated in inhibitory interneurons reduces GABA release, overexciting granule cell layer glutamatergic neurons. This may be a key factor in increasing extracellular glutamate, ultimately causing NSC apoptosis.

### 3.2 Interneuron Accumulation of GSK‐3β Disrupts Vesicular Function and Impairs GABA Transmission and Disinhibited Local Neural Circuits

The accumulation of GSK‐3β impairs GABAergic neurotransmission, resulting in the disinhibition of local excitatory neurons. Dysfunctional GABAergic transmission and glutamatergic neuron hyperexcitability can shift neural precursor cell fate toward glial lineage differentiation (Obernier et al. 2018; Zheng et al. 2020). Local excitatory stimuli not only regulate dendritic development and circuit integration in immature neurons but also directly influence neural precursor cells by modulating fate‐determining genes (such as Hes1 and NeuroD1), thereby shaping their differentiation trajectory (Deisseroth et al. 2004; Dong et al. 2019). Dysregulated GABAergic transmission can directly modulate the fate determination of NSCs and, through the activation of local neural networks, indirectly impair the process of neurogenesis.

To understand the mechanisms underlying the hTau‐induced GABA reduction, we measured the factors regulating GABA synthesis, release, uptake, and transport, and enhanced phosphorylation of numerous presynaptic‐associated proteins was found. The phosphorylation of calcium channel proteins and presynaptic vesicular release‐related proteins was significantly enhanced. Notably, the CACNA1 protein (Catterall 2011; Ertel et al. 2000), which encodes the presynaptic Cav2.2 (N‐type calcium channel), and syntaxin, a key component of the SNARE complex (Gerber et al. 2008), exhibited marked increases in phosphorylation. GSK‐3β is a ubiquitous phosphokinase that performs a variety of functions in neurons through substrate phosphorylation (Clayton et al. 2010). During vesicle release, GSK‐3β can inhibit the interaction with its synapse‐related proteins, thereby affecting P/Q‐type voltage‐dependent Ca^2+^ channels (VDCC) activity (Zhu et al. 2010). The present study revealed that overexpression of GSK‐3β in GABAergic interneurons led to enhanced presynaptic VDCC and SNARE protein phosphorylation. Notably, it has been shown that phosphorylation of N‐ethylmaleimide‐sensitive factor connector complex (SNARE) component proteins has been demonstrated to inhibit their binding (Matos et al. 2003; Snyder et al. 2006). Furthermore, phosphorylation of VDCC has been shown to further weaken the binding affinity between SNARE‐component proteins, thus impairing vesicle release. By increasing the phosphorylation of SNARE proteins and VDCCs, GSK‐3β establishes a reciprocal reinforcement between these events, resulting in the inhibition of synaptic vesicle release. Modifications related to diverse synaptic transmission mechanisms as well as those associated with synaptic plasticity have also been identified through phosphoproteomics. Nevertheless, we contend that this phenomenon is an indirect consequence of dysregulation in GABA transmission rather than a primary factor.

### 3.3 Strengthening GABAergic Transmission Rescued AHN Deficits

A progressive loss of neurons is observed in individuals diagnosed with AD (Ihara et al. 2018). The maintenance of a viable stem cell population and repair of degenerative AHN have emerged as pivotal strategies for the treatment of AD. The transplantation of neural stem cells represents an efficacious method for increasing the number of neurons within the brain (Kim et al. 2015; Yue et al. 2015). However, the complex internal environment of the AD brain complicates efforts to determine the differentiation pathways of transplanted stem cells, making it challenging for differentiated neurons to integrate into the existing neural network. GABA, the major inhibitory neurotransmitter in the adult brain, initially exerts an excitatory action on newborn neurons owing to their high cytoplasmic chloride ion content and then promotes their synaptic formation and dendrite development (Ge et al. 2006). GABA receptors are classified into two main types: ionotropic GABAA receptors and metabolic GABAB receptors. Functional GABAA receptors are expressed on NSCs and migrating neurons, whereas GABAB receptors play a role in regulating both proliferation rates and the differentiation process among maturing neurons (Sibbe and Kulik 2017). Activation of GABAA receptors located on NSC membranes induces cellular hyperpolarization, thereby maintaining a quiescent state through inward flow of chloride ions [Cl^−^] (Andang et al. 2008). Furthermore, activation of GABAA receptors has been shown to enhance self‐renewal capabilities while reducing apoptosis rates of type II IPCs. Direct administration of neurotransmitter GABA has demonstrated efficacy in mitigating Aβ toxicity in young AD model mice (Sun et al. 2012). Additionally, transplantation studies involving GABAergic neurons and their precursor cells have proven effective at restoring brain rhythm in AD mouse models (Magdalena et al. 2018; Tong et al. 2014). The present study has revealed that gaboxadol is capable of maintaining the resting state of NSCs and preserving the stem cell pool that supports AHN. A promising strategy to enhance GABA transmission involves increasing both the number and activity of GABA interneurons or improving the functionality of GABA receptors. This approach also presents a potential therapeutic pathway for AHN injury in AD mouse models.

## 4 Conclusion

The present study revealed that overexpression of GSK‐3β in GABAergic interneurons impairs AHN and induces NSCs apoptosis by deteriorating GABA transmission, and administration of GABAergic agonists is efficient in preserving AHN. These results revealed a novel mechanism underlying the AHN impairments in AD and provided new targets for pro‐neurogenic therapies.

## 5 Methods

### 5.1 Antibodies, Virus and Chemicals

The details of the primary antibodies, viruses, and chemicals employed in the present study are listed in Table S1.

### 5.2 Animals

The 3×Tg‐AD [129S4.Cg‐Tg (APPSwe, tauP301L)1Lfa Psen1tm1Mpm/LfaJ] mice and wild‐type 129 (129S1/SvImJ) mice, vGAT‐cre mice, and Nestin‐GFP mice were obtained from Jackson Laboratory. The vGAT‐cre: Nestin‐GFP mice were generated by crossbreeding vGAT‐cre mice with Nestin‐GFP mice. All mice were housed under standard conditions with a 12‐h light/dark cycle, with food and water provided ad libitum. Only male mice weighing 20–30 g were used in all experiments. All animal studies adhered to the guidelines established by the Ethics Committee of Binzhou Medical University.

### 5.3 Viruses and Stereotaxic Injection

For virus injection, mice were anesthetized with a combination of ketamine (100 mg/kg) and xylazine (50 mg/kg), and then fixed in a stereotaxic apparatus (RWD, China). The scalp was sterilized with iodophors and incised along the midline of the skull. Bilateral holes were then drilled, allowing for the delivery of a total of 500 nL of virus into each site within the dentate gyrus (posterior 1.9 mm, lateral ±1.1 mm, and ventral 2.0 mm relative to bregma). This was accomplished through the use of an automatic microinjection system (LEADFLUID, China) at a rate of 50 nL/min. Subsequently, the needle was maintained in position for 10 min before being gradually retracted. The incision was then sutured and sterilized with iodophors. The mice were placed in a thermos tank to induce anesthesia.

### 5.4 In Vivo Optic Fiber Recording

Optic fiber cannulas (NA = 0.37; Inper, China) were implanted into the SGZ of mice (AP1.9, LM ± 1.1, DV 2.0) via analogous procedures to those previously described above for tetrode implantation. Prior to the initial recording session, each mouse was handled and acclimated to the T‐maze environment for 10 min per day over a period of three consecutive days. The GCaMP6s or iGABASnFR signals were recorded using a Fiber Photometry system (Thinker Tech, China), with the LED power set at 65 mW. During the recording sessions, mice were permitted to move freely within the T‐maze. The data were analyzed using MATLAB, and the ΔF/F was calculated according to the following formula: (*F* − *F*_0_)/(*F*_0_ − *F* offset) × 100%. A threshold of 5% Δ*F*/*F* was established for quantifying calcium or GABA responses. Individuals with off‐target fiber end locations were excluded from the subsequent analyses.

### 5.5 AHN Evaluation

Fifty micrometers brain sections were obtained using a vibrating microtome (CM3050S; Leica, Germany). This study focused on the evaluation of the AHN in the dorsal DG, specifically spanning approximately from AP −1.7 to −2.5. Immunofluorescent images were acquired by scanning a z series stack at a 3 μm interval throughout the entire 50 μm thickness. BrdU (B9285; Sigma‐Aldrich) was dissolved in 0.01 M PBS (10 mg/mL) and administered via intraperitoneal injection at a dosage of 50 mg/kg for five consecutive days prior to euthanasia to assess cell proliferation. Cell quantification was performed following previously established methodologies (Liu et al. 2020). In brief, every fifth section was stained and then counted by an experimenter who was blinded to the animal groupings. The counts obtained were multiplied by five and summed to represent the total number of cells within the dorsal DG. Cell numbers in both left and right DG were counted separately but averaged for analysis. The dendrite length and complexity of ROV‐GFP‐labeled newborn neurons were assessed using Sholl analysis with the Simple Neurite Tracer plugin (Longair et al. 2011) and ImageJ (Fiji) software (Schindelin et al. 2012) Dendrite spines of ROV‐GFP‐labeled neurons were imaged under a 100× oil immersion lens. Only secondary dendrites were analyzed in this study.

### 5.6 Western Blotting and Dot Blotting

Western blotting and dot blotting were conducted following established protocols from previous studies (Li et al. 2017; Liu et al. 2020). In brief, the mice were euthanized using an overdose of ketamine and xylazine, after which the dentate gyrus was isolated as described by Hagihara Toyama et al. (2009). Proteins were extracted using RIPA buffer (P0013B; Beyotime). The protein concentration was quantified using the BCA method. An equal amount of total protein was loaded for each sample, separated by 10% SDS‐polyacrylamide gel electrophoresis, and subsequently transferred onto nitrocellulose membranes. The membranes were blocked with 5% defatted milk for 1 h at room temperature before being incubated overnight at 4°C with primary and secondary antibodies (1:1000–1:2000), in succession. Following washing with TBST, bands were visualized using an Alphalmager System (FluorChem HD2; Protein Simple). The resulting images were then analyzed using Image J software (Fiji).

### 5.7 Immunofluorescence

Mice were anesthetized using a combination of ketamine (100 mg/kg) and xylazine (50 mg/kg), followed by transcardial perfusion with normal saline, succeeded by 4% paraformaldehyde (PFA). Brains were subsequently extracted and post‐fixed for an additional 48 h. Thereafter, the tissue was sliced at 50 μm using a vibrating microtome (VT1000S; Leica, Germany). Sections were permeabilized in phosphate buffer containing 0.5% Triton X‐100 for 30 min, blocked with 3% BSA for another 30 min, and then incubated overnight at 4°C with primary antibodies. Subsequently, the brain sections were washed in PBS and incubated with Alexa Fluor‐conjugated goat secondary antibodies (1: 500; Abbkine), respectively, for 1 h at room temperature. Images were obtained using a laser‐scanning confocal microscope (Stellaris 5; Leica, Germany).

### 5.8 Phosphoproteomic Analysis

The mice were euthanized by cervical dislocation, and their brains were promptly excised for further analysis. The DG tissues were isolated on ice following the methodology previously described. Subsequently, the samples were ground in liquid nitrogen and then dissociated by sonication in a buffer containing 8 M urea and a protease inhibitor cocktail at a concentration of 1%. Following this, the samples were collected after centrifugation at 4°C and 12,000 rpm for 10 min. The protein concentrations were determined using the bicinchoninic acid (BCA) assay (Beyotime), as outlined previously.

For mass spectrometry analysis, protein solutions were reduced with dithiothreitol (5 mM) for 30 min at 56°C and subsequently alkylated with iodoacetamide (11 mM) for 15 min at room temperature in the absence of light. Following this, the samples were diluted with triethylammonium bicarbonate to ensure that the final concentration of urea was maintained below 2 M. Trypsin (Promega) was added at a ratio of 1:50 (enzyme to protein) for the initial digestion, which was carried out overnight. The second digestion utilized a ratio of 1:100 and lasted 4 h. Afterward, the peptides were desalted using a Strata X C18 SPE column (Phenomenex), vacuum‐dried, and reconstituted in 0. The samples were then labeled using a TMT kit (Thermo Fisher) and fractionated by high pH reverse‐phase HPLC employing an Agilent 300Extend C18 column (5 mm particles, 4.6 mm ID, 250 mm length). The mobile phase comprised of 5 M TEAB. The enrichment of phosphopeptides was achieved through immobilized metal affinity chromatography (IMAC) microspheres. Mass spectrometry data were collected using a Q Exactive Plus Hybrid Quadrupole‐Orbitrap mass spectrometer coupled with an EASY‐nLC 1000 liquid chromatography pump (Thermo Fisher Scientific). Subsequently, the data underwent processing via the Maxquant search engine (v.1.5.2.8). The resulting mass spectra data were subjected to a search against the SwissProt Mouse database concatenated with a reverse decoy database.

The expression levels of each phosphopeptide across all six samples were normalized horizontally and converted into log_2_ values. The mean values were calculated as Mm (mCherry group) or Mt (GSK‐3β group). Two‐tailed *t*‐tests were employed to evaluate the differences between the mCherry and GSK‐3β groups. For each phosphopeptide, a *p*‐value of less than 0.05 coupled with a ratio of Mt/Mm greater than 1.2 was deemed indicative of significant upregulation, whereas a *p*‐value of less than 0.05 and a ratio of Mt/Mm less than 1/1.2 was considered indicative of significant downregulation.

The phosphorylated proteomic data underwent bioinformatics analysis to facilitate Gene Ontology (GO) annotation. To achieve this, UniProt IDs obtained from the UniProt‐GOA database were converted to map them to GO IDs. In cases where some identified proteins in mass spectrometry (MS) lacked prior annotation in the UniProt‐GOA database, InterProScan software was utilized to assign their GO functional annotations based on protein sequence alignment methods. Subsequently, these proteins were classified according to their biological processes in accordance with the GO annotations. The enrichment analysis for differentially expressed proteins was conducted relative to all identified proteins using a two‐tailed Fisher's exact test. A corrected *p*‐value of less than 0.05 is deemed statistically significant concerning the GO analysis.

### 5.9 Contextual Pattern Separation

Mice were handled for three consecutive days before test and then conditioned by a 2 s, 65 mA footshock in context A (i.e., the standard fear conditioning chamber) after 180 s of free exploring in the chamber on day 1. The next day, mice were tested for fear generalization for 180 s, in turn, in context A and a very distinct context (context B, a 45 × 45 × 45 cm open field with Plexiglas floor and walls), both without footshock. Freezing behaviors of each mouse were recorded and analyzed online by an XR‐XC404 system (Softmaze, China).

In subsequent days, mice were trained through repeated trials to discriminate a pair of similar contexts with much more common features (i.e., context A and C). Context A was paired with footshock and characterized by an additional 65 dB white noise, and odorless 5% sodium hydroxide solution was used to clean the chamber between animals. By contrast, context C was paired with green background lighting, but without footshock. A pan filled with 0.25% benzaldehyde in alcohol was placed under the grid floor of the chamber in each trial to avoid the interference between different animals (Nakashiba et al. 2012). The order of context A and C each day during the training followed a sequence in pseudorandom. Freezing behavior was defined as behavioral immobility except for movement necessary for respiration. Discriminative scores between context A and C were calculated as the freezing time (A − C)/(A + C) and averaged for every 2 days as the mean score in each block.

### 5.10 Statistical Analysis

Data are presented as means ± SEM, unless otherwise specified. All data were analyzed and plotted using GraphPad Prism 8 software. The statistical analysis was performed utilizing unpaired or paired two‐tailed *t*‐tests, ANOVA (one‐way, two‐way, or repeated measures), and post hoc Tukey's multiple comparisons test, as detailed in the figure legends. The threshold for statistical significance was established at *p* < 0.05.

## Author Contributions

F.L. designed the research; J.‐J.C., X.‐L.L., Z.‐M.Z., Q.Z., and Y.‐F.L. performed the experiments, F.L., S.‐H.L., and H.‐L.Q. analyzed the data, and F.L. and J.‐J.C. wrote the manuscript. Y.S., J.‐M.L., and J.Y. helped with the experiments and made constructive suggestions.

## Conflicts of Interest

The authors declare no conflicts of interest.

## Supporting information


Data S1.

## Data Availability

The data used to support the findings of this study is available from the corresponding author upon request.
