# Supplementary material for: Accumulation of GSK‐3β in Interneurons Impairs Adult Hippocampal Neurogenesis by Inhibiting GABAergic Transmission
Source: Aging Cell. 2025 May 26;24(8):e70115. doi: 10.1111/acel.70115 (PMC12341769; doi:10.1111/acel.70115)
Supplement: Supplementary file 1 — Data S1. [file ACEL-24-e70115-s001.docx]

**Supplementary**

**Table S1**

| REAGENT or RESOURCE | SOURCE | INENTIFIER |
| --- | --- | --- |
| Antibodies |  |  |
| Anti-β-actin | Abcam | Cat#ab6276 |
| Anti- GSK-3β-tyr216 | Millipore | Cat#05-413 |
| Anti-BrdU | Bio-Rad | Cat#MCA2483 |
| Anti-DCX | Abcam | Cat#ab18723 |
| Anti-Flag | Proteintech | Cat#20543 |
| Anti-GAD67 | Millipore | Cat#MAB5406 |
| Anti-GAD65 | Abcam | Cat#ab26113 |
| Anti-GAT1 | Proteintech | Cat#28488 |
| Anti-GFAP | Cell Signaling | Cat#3670 |
| Anti-MCM2(BM28) | BD Biosciences | Cat#610700 |
| Anti-NeuN | Millipore | Cat#MABN140 |
| Anti-NeuroD1 | Abcam | Cat#ab213725 |
| Anti-SOX2 | Abcam | Cat#ab97959 |
| Anti- Cleaved-casepase3 | Abcam | Cat#ab2302 |
| Anti-Synapsin1 | Abcam | Cat#ab32127 |
| Anti-Synaptotagmin | Abcam | Cat#ab13259 |
| Anti-Gerphrin | Abcam | Cat#ab181382 |
| Anti-VAMP2 | Proteintech | Cat#10135 |
| Anti-VGAT | Santa Cruz | Cat#sc-393373 |
| Anti-GABA | Sigma | Cat#A2052 |
| Anti-GABA_A_R-α1 | Abcam | Cat#ab151573 |
| Anti-GABA_A_R-β1 | Proteintech | Cat#28425 |
| Anti-GABA_A_R-γ2 | Abcam | Cat#ab288564 |
| HRP-conjugated Goat Anti-Mouse IgG | Abbkine | A21010 |
| HRP-conjugated Goat Anti- Rabbit IgG | Abbkine | A21020 |
| Dylight 488. Goat Anti-Mouse IgG | Abbkine | Cat#A23210 |
| Dylight 649. Goat Anti-Mouse IgG | Abbkine | Cat#A23610 |
| Dylight 594, Goat Anti-Mouse IgG | Abbkine | Cat#A23410 |
| Dylight 405, Goat Anti-Rabbit IgG | Abbkine | Cat#A23120 |
| Dylight 649, Goat Anti-Rabbit IgG | Abbkine | Cat#A23620 |
| Dylight 594, Goat Anti-Rabbit IgG | Abbkine | Cat#A23420 |
| Dylight 488, Goat Anti-Rabbit IgG | Abbkine | Cat#A23220 |
| Virus Strains |  |  |
| rAAV-EF1α-DIO-GSK-3β-mCherry-WPRE | BrainVTA | Cat#PT-6555 |
| rAAV-EF1α-DIO -mCherry-WPRE-hGH pA | BrainVTA | Cat#PT-0013 |
| rAAV-CamkⅡa-Gcamp6s-WPRE-hGH pA | BrainVTA | Cat#PT-0110 |
| AAV-CamkⅡa-EGFP-WPRE- hGH pA | BrainVTA | Cat#PT-0290 |
| rAAV-hSyn-iGABASnFR-WPRE-SV40 pA | BrainVTA | Cat#PT-1249 |
| Prov-U6-EF1α (S)-EGFP-3×FLAG-WPRE | OBio | CN889 |
| Chemicals |  |  |
| gaboxadol hydrochloride | MCE | Cat#HY-10233 |
| Clozapine N-oxide (CNO) | Sigma | Cat#C0832 |
| DAPI | Beyotime | Cat# C1002 |
| 5-Bromo-2-Deoxyuridine (BrdU) | Sigma | Cat#B9285 |

**
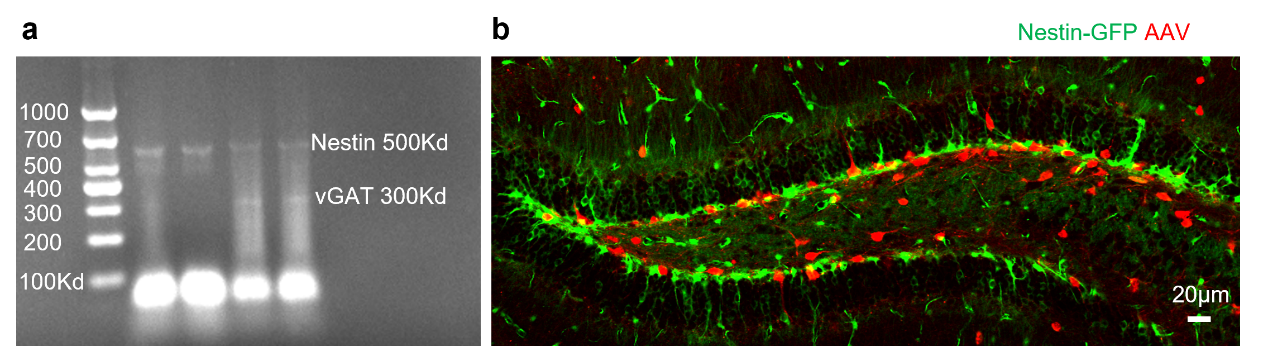
**

**Figure S1. The display of the genotype of nestin: vGAT-cre mouse.** (**a**) Nestin:VGAT-cre transgenic mice co-express Nestin, a neural stem cell marker, and vGAT, a GABA-transporting protein. (**b**) The AAV-EF1a-DIO-GSK-3β-mCherry virus can only be expressed in the inhibitory interneurons of vGAT - cre mice, which simultaneously express the nestin-GFP protein.
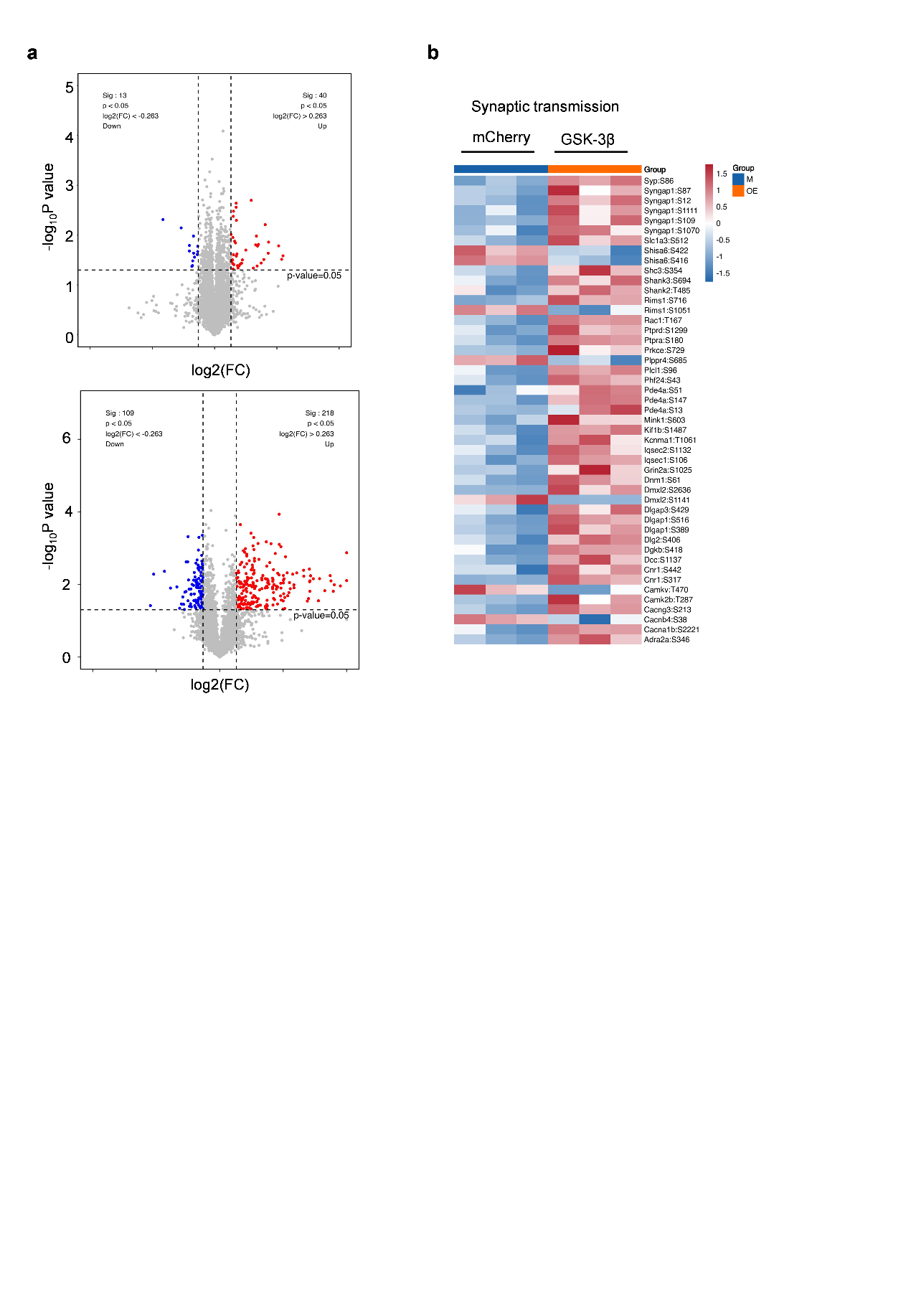


**Figure S2. Specific Overexpression of GSK-3β Altered Synaptic Transmission.** (**a**) Proteomic and phosphoproteomic analyses on proteins extracted from the DG region. (**b**) An integrated analysis of both the proteome and phosphorylated proteome identified proteins and phosphorylation sites implicated in synaptic transmission identified through.

**
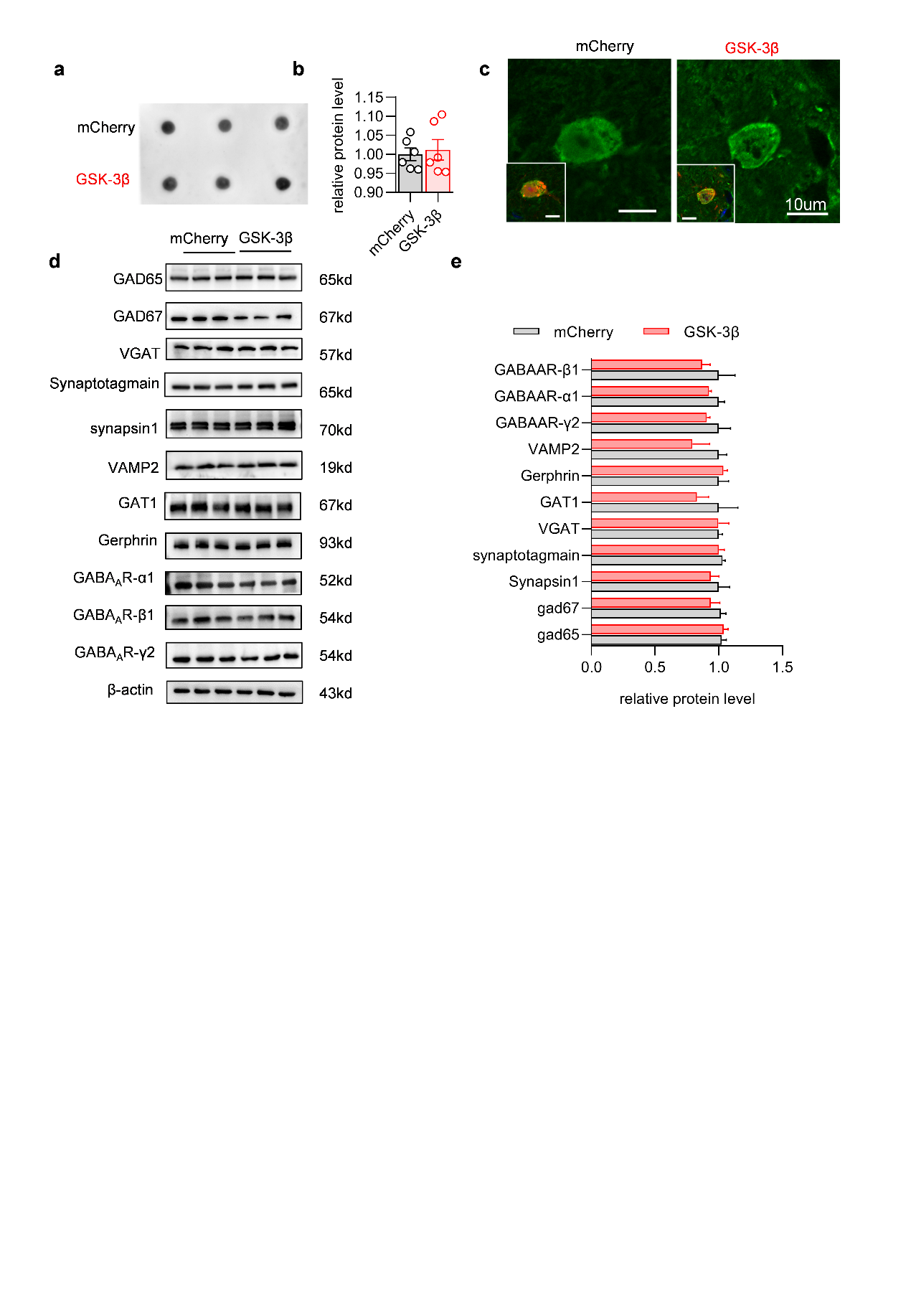
**

**Figure S3. Specific Overexpression of GSK-3β Did Not Affect the Expression of Key Proteins Involved in** **GABA Metabolism.** (**a, b**) The expression of GABA in DG remained unchanged detected by dot blotting. Unpaired t tests, n=6 mice in each group. (**c**) Representative immunofluorescence images demonstrate that intracellular levels of GABA are unaffected in interneurons overexpressing GSK-3β. Scale bar, 10μm. (**d, e**) Specific overexpression of GSK-3β in GABAergic interneurons did not alter the expression of proteins involved in the synthesis, transport, release and reuptake of GABA within the DG region. Data were normalized to β-actin and presented as mean values related to mCherry control group for each protein. Unpaired t tests, n = 4–6 mice.


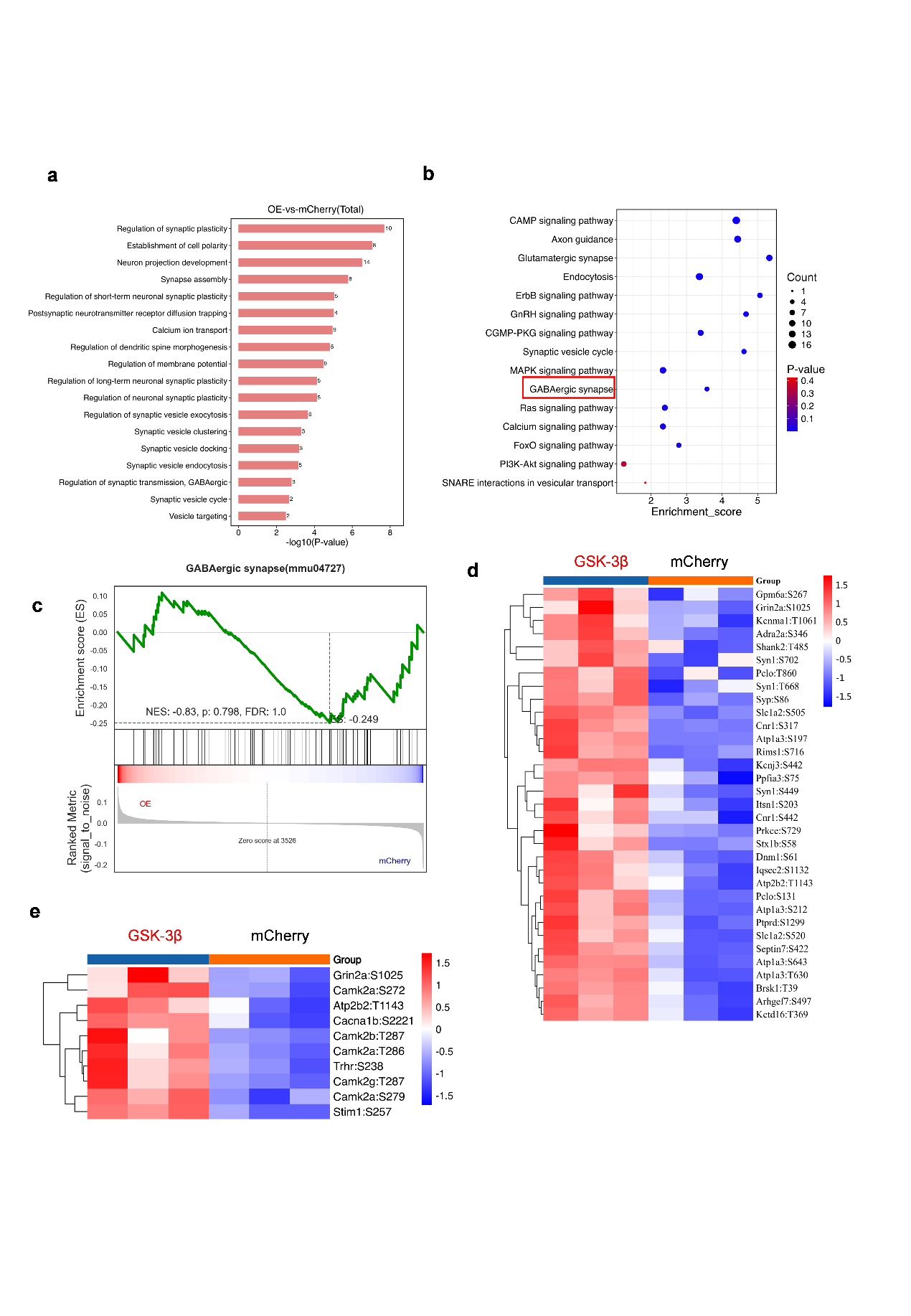


**Figure S4. Specific Overexpression of GSK-3β** **Altered Phosphorylation at Numerous Sites.** (**a**) Altered phosphorylation sites are implicated with a variety of biological processes. (**b, c**) A marked reduction was observed within the gabaergic synaptic pathway following these alterations. (**d**) Increased phosphorylation events of presynaptic proteins were documented. (**e**) Increased phosphorylation events involving calcium signaling pathways were also documented.
